# Anytime collaborative brain–computer interfaces for enhancing perceptual group decision-making

**DOI:** 10.1038/s41598-021-96434-0

**Published:** 2021-08-20

**Authors:** Saugat Bhattacharyya, Davide Valeriani, Caterina Cinel, Luca Citi, Riccardo Poli

**Affiliations:** 1 Brain Computer Interfaces and Neural Engineering Laboratory, School of Computer Science and Electronic Engineering, University of Essex, Wivenhoe Park, Colchester, CO4 3SQ UK; 2 Department of Otolaryngology, Head and Neck Surgery, Massachusetts Eye and Ear, Harvard Medical School, 243 Charles St, Boston, MA USA; 3 School of Computing, Engineering and Intelligent Systems, Ulster University, Northland Road, Londonderry, BT48 7JL UK

**Keywords:** Computational science, Scientific data

## Abstract

In this paper we present, and test in two realistic environments, collaborative Brain-Computer Interfaces (cBCIs) that can significantly increase both the speed and the accuracy of perceptual group decision-making. The key distinguishing features of this work are: (1) our cBCIs combine behavioural, physiological and neural data in such a way as to be able to provide a group decision at any time after the quickest team member casts their vote, but the quality of a cBCI-assisted decision improves monotonically the longer the group decision can wait; (2) we apply our cBCIs to two realistic scenarios of military relevance (patrolling a dark corridor and manning an outpost at night where users need to identify any unidentified characters that appear) in which decisions are based on information conveyed through video feeds; and (3) our cBCIs exploit Event-Related Potentials (ERPs) elicited in brain activity by the appearance of potential threats but, uniquely, the appearance time is estimated automatically by the system (rather than being unrealistically provided to it). As a result of these elements, in the two test environments, groups assisted by our cBCIs make both more accurate and faster decisions than when individual decisions are integrated in more traditional manners.

## Introduction

Making decisions—either individually or in group—is an important aspect at all levels of everyday life. Decisions (for example made by government, military or hospital management) can be highly critical in nature, with mistakes possibly resulting in extremely adverse outcomes, including loss of lives. Often, decisions have to be made with limited amounts of information, or indeed too much information for any single person to process in a meaningful manner, hence involving a high degree of uncertainty. In such cases, suboptimal decisions are likely.

Decision-making under uncertainty has been extensively studied in the fields of, for example, neuroeconomics^1^, decision theory and behavioural economics^2,3^. In all areas, what has emerged through decades of research is that decisions in everyday life are dominated by a practical approach—rather than strictly rational—based on heuristics and biases^4–6^. Models for decision-making under uncertainty include those in the area of situational awareness, which typically take into account behavioural and cognitive factors, such as perceptual overload, attention and vigilance fluctuations, working memory limitations, trust and communication, and often include artificial agents support for enhancing situation awareness and, ultimately, decision making^7–11^.

In this paper, we focus on decision making based on perceptual judgement in situations where time is critical, and where decisions are binary (i.e., discrimination between two types of target), and are either correct or incorrect, and propose a system that increases the likelihood of correct outcomes. In the two experiments described here, decisions are based on sensory evidence coming from the external environment, which is intrinsically noisy, making decision prone to errors. In both experiments, participants are asked to make the perceptual decisions as rapidly as possible. In the second experiment, however, participants have also a choice of delaying their response in order to acquire more information and, therefore, increase the likelihood of a correct decision, which, however, also increases the cost associated to not deciding on time.

Typically, decisions are associated with varying degrees of confidence, defined as the evaluation of one’s own performance. The degree to which confidence is accurate (in the sense that it is a reflection of the probability of the decisions being correct) is known as *metacognitive accuracy*^12,13^. Confidence tends to be correlated with the accuracy of decisions, although sometimes not very strongly, and it may also be uncalibrated (e.g. biased towards overestimating or underestimating the true probability of the decision being correct)^14–17^.

In difficult decision tasks where individuals tend to present low accuracy and correspondingly low metacognitive accuracy, groups usually make better decisions than individuals (e.g., wisdom of crowds)^18,19^. However, there are circumstances in which group decision-making can be suboptimal^20,21^ or even disadvantageous^22–24^. Flaws can be caused by, for example, difficulties in coordination and interaction between group members, reduced member effort within a group, strong leadership, group judgement biases, and so on^25–27^.

One way to enhance the performance of groups is to take into account the decision confidence that accompanies each individual opinion, usually reported by the members themselves^24,28–31^. For instance, weighing the opinion of each member by their respective confidence^30,32^ makes the group’s decision more dependent on individuals who have reported high confidence, which tends to improve accuracy, particularly in the presence of *tie decisions*. In such cases, ties can be resolved in favour of decisions associated with greater collective confidence. This approach may also be effective in situations where a minority of group members reports high confidence for a particular choice, while the majority reports low confidence for another choice, as in such cases, it is more rational to trust the most confident members rather than the majority. When using confidence weighting, the group final decision could be the choice of the more confident minority.

However, many circumstances exist where there is weak or no correlation between decision confidence and the accuracy of the decision^19,33^. Factors that may affect the degree of association between accuracy and confidence, irrespective of the tasks include individual attributes such as personality, gender, or culture^19^, and the time of day when the decision confidence was measured^34^. There are also cases where there is a negative correlation between confidence and accuracy where incompetent individuals can be exceptionally overconfident^35^, which can lead to potential decision-making disasters. Also, there are many situations where rapid decisions are required, and waiting for each user to express their confidence after their decisions is not feasible. Brain-Computer Interfaces (BCIs) offer a potential solution to these problems.

Research on BCIs has traditionally had the goal of improving the quality of life of people with severe disabilities who would be otherwise unable to communicate or control devices such as prostheses, wheelchairs, mouse pointers, etc.^36–49^. However, more recently it has been possible to extend the applications of BCIs to able-bodied individuals to enhance or complement existing functions. This is done, for example, in the areas of: (a) *neuroergonomics*, which uses the neural and cognitive activity underpinning human performance to design systems that allow humans to perform in a safer and more efficient way in everyday tasks and in the work-place^50,51^; (b) *passive BCIs*^52–55^, which monitor spontaneous (i.e. not directly triggered by the BCI itself) brain activity of users performing everyday activities, and react in ways that facilitate such activities for the users; and (c) *collaborative BCIs* (cBCIs), where the brain activities of multiple users are integrated to achieve a common goal^33,56–67^. The last form, cBCIs, offer a solution to the problem of improving group decision-making.

Because brain signals differ widely from person to person, normally cBCIs do not integrate brain activity of multiple users at that level. Instead, typically they give each user a decoder of their decisions, which is a classifier trained to best recognise such decisions for that user^58–61,68,69^. Group decisions are then formed by adding up the analogue outputs (the decision function value) of each classifier, so that outputs further away from the decision boundary have higher influence on the outcome. Research has shown that groups assisted by this form of cBCI performed better than single users reporting decisions via key presses. However, the performance of such groups was substantially lower than that obtained from an equally sized group using standard majority voting to make decisions. The fundamental reason for this is that the error correction benefits of the wisdom of crowds were reduced by the imperfect interpretation of the user intentions associated with even the best BCIs. On the contrary, in groups signalling decisions with key presses, user intentions are almost never misinterpreted.

**Figure 1:**
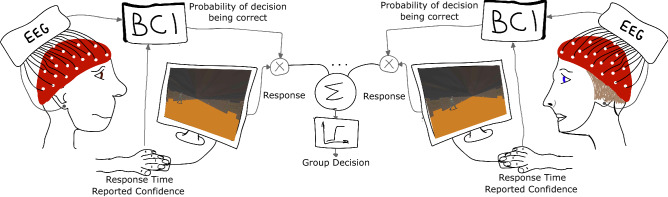
An illustration of our developed form of cBCI. Here, a combination of response time, reported confidence and neural signals are used to estimate the probability of being correct on a decision. Finally, the group decision is made by the aggregation of weighted responses.

A quantum leap in performance was obtained with a particular form of cBCIs that we developed^70^, which we call *hybrid cBCIs* because they use a combination of neural, behavioural and physiological measurements. Here the objective was not to infer user intentions (these were reported by key presses), but to estimate the objective confidence (i.e., the true probability of being correct) of the members of a decision-making group on a decision-by-decision basis. This confidence was then used as a weight for the decision of the corresponding team member when aggregating individual contributions to form group decisions. An illustration of our cBCI architecture is shown in Fig. 1.

**Figure 2:**
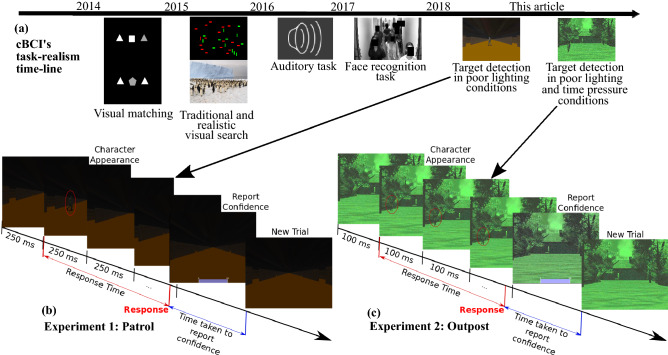
(**a**) Progress made to date in the development of collaborative BCIs for realistic, decision-making tasks. (**b**,**c**) Examples of video sequences in a single trial of (**b**) Experiment 1: Patrol and (**c**) Experiment 2: Outpost. The character appears only in the second frame of the example followed by a response reported by the participant (marked in red). After the response, the participant indicates his/her degree of confidence, which is shown as 100 in this example.

Over the years, we have tested this cBCI architecture on a variety of tasks of increasing realism, including visual matching tasks^71^, visual search with simple shapes^72,73^, visual search with realistic stimuli^33,74^, face recognition^75,76^ and threat detection with video stimuli^77–79^. In all cases, decisions supported by the cBCIs were superior (both in terms of accuracy and speed) in comparison to their non-BCI counterparts (standard majority or weighing decisions using self-reported confidence) when *comparing equally sized groups*. A timeline of the implementing cBCI from traditional to realistic decision-making task is illustrated in Fig. 2(a).

In this paper we focus on cBCIs integrating physiological, neural and self-reported data across multiple participants to produce both faster and more accurate group perceptual decisions. Specifically, we make the following contributions.

Firstly, we present the first *anytime* cBCI. Like other anytime algorithms^80^, our cBCI makes an approximate decision always available, but the longer one can wait, the better the decision gets. This property is particularly important in domains (e.g. in military, medical or financial contexts) where there is time pressure to reach a decision as the risks associated with further delaying it become rapidly greater than the risks of an incorrect decision.

Secondly, we apply our cBCIs to two realistic scenarios of military relevance (patrolling a dark corridor and manning an outpost at night where users need to identify any unidentified characters that appear) in which decisions are based on information conveyed through video feeds. Both the complexity of the scenarios and the use of video feeds are unique features (and presented unique challenges).

Finally, we have simulated a real-life situation where users watch continuous video feeds and independently decide when a relevant event has occurred which requires a decision. Here, one only knows for sure when an individual completes the process of making a decision (as decisions are signalled by a button press), but not what triggered it and when. Here we have provided the cBCI with the ability to automatically detect significant changes in the video stream prior to the response, thereby making it possible for it to approximately work out the timing of triggering events. The timing of the trigger is important to be able to reconstruct the response time (RT), which has proven to be an important correlate of the probability of the decisions being correct in both the psychophysiology literature and in our previous work on cBCIs for decision-making. Trigger timing is also important because it makes it possible to extract information from stimulus-locked Event-Related Potentials (ERPs), which are normally impossible to extract from video feeds, unless the videos have been previously manually labelled.

### Results

#### Tasks

We have tested our cBCI system in two decision-making experiments of military relevance. Experiment 1 presented video sequences representing the viewpoint of a soldier walking along a poorly lit corridor with doors on either side. Computer-generated characters would suddenly appear from doors (see Fig. 2(b)). Experiment 2 simulated a situation where a soldier is at an outpost at night and a computer-generated character starts walking towards it (see Fig. 2(c)). Time pressure and a reward/penalty system were included to simulate a situation where both erroneous and slow decisions may have had negative consequences.

In both scenarios, participants had the task of reporting whether the characters appearing were wearing a helmet or a cap by pressing a mouse button. Both experiments received the UK Ministry of Defence (MoD)’s ethical approval in July 2017 and were performed in accordance with relevant guidelines and regulations. The tasks for both experiments were designed after consultation with the MoD. Decision confidences derived by the cBCI from neural and behavioural features were used in combination with their corresponding decisions to reach a final group consensus for each trial. Participants performed the experiments individually, and group decisions with groups of sizes two to ten were performed post-hoc by considering all possible combinations of participants.

**Figure 3:**
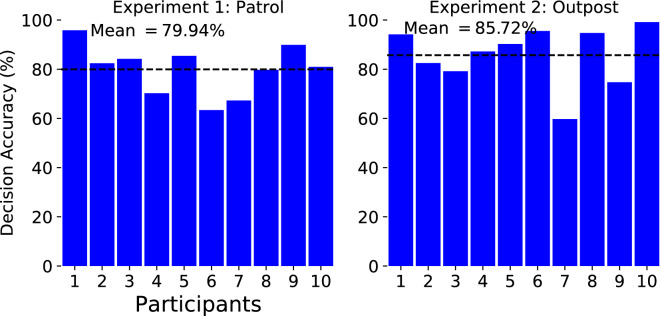
The individual accuracies of the ten participants for Experiments 1 and 2^79^.

#### The tasks are difficult for individual decision makers

Figure 3 shows the individual accuracies of the participants in Experiments 1 (left) and 2 (right). Due to the poor lighting conditions, the tasks are relatively difficult, the average decision accuracies (dashed line in the figures) being $79.94\% \pm 9.67\%$ and $85.72\%\pm 11.42\%$(first reported in^79^), respectively. Experiment 1 is difficult because of the poor lighting conditions and because the character appears on the screen for only 250 ms and at random locations. Experiment 2 also has very poor lighting conditions but it is slightly easier as the character stays on the screen for much longer and becomes progressively bigger, which makes it possible for participants to foveate and wait until there is enough detail to be reasonably sure of their response. A part of our objective for this study is to show the improvement in group decision-making over individual decision-making (as shown in Fig. 5).

#### ERP analysis shows differences in brain activity for correct and incorrect decisions

**Figure 4:**
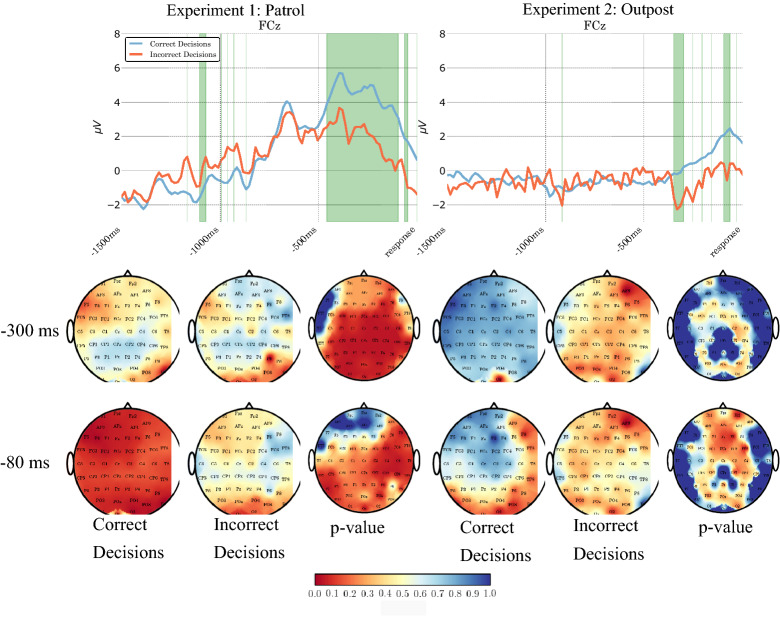
The plots on the top are grand averages of the response-locked Event Related Potentials at FCz channel location for correct (in blue) and incorrect (in red) decisions for Experiments 1 and 2. Regions shaded in green are when there is a significant difference ($\hbox {p}<0.05$; Wilcoxon two-tailed signed rank test) between the correct and incorrect ERPs. The topographical scalp maps at the bottom represent the grand averages for correct and incorrect decisions and corresponding *p*-values obtained from the Wilcoxon two-tailed signed ranked test over all electrode locations at 300 ms and 80 ms before the response for Experiments 1 and 2. The colour map at the bottom is a scale for *p* values.

We have examined the Event Related Potentials (ERPs) associated with correct and incorrect decisions made for all participants. Figure 4 (top plots) shows the response-locked grand averages of the ERPs at the FCz electrode location for correct and incorrect trials. Green shading marks the regions where the Wilcoxon signed-rank test indicated that differences between correct and incorrect trials are statistically significant. For Experiment 1, it is apparent that differences are significant for approximately 500 ms preceding the response. For Experiment 2, differences are present in the period preceding the response too, but they are statistically significant only in much smaller time intervals than for Experiment 1.

The situation is similar for many other electrode sites, as one can see in the scalp maps in Fig. 4 (bottom) which represent the activation maps during correct and incorrect decisions (grand averages) and p-value of the Wilcoxon signed-rank test that compared the grand averages of the correct and incorrect responses at 300 ms and 80 ms before the response.

The differences in the patterns of brain activity recorded in the two experiments are most likely due to the fact that in Experiment 1 uniformed characters on which the decision is based appear suddenly and for a very short time and then disappear, while in Experiment 2 they appear initially very small and then progressively become bigger and bigger as they walk towards the outpost. So, there is not a very well-defined event that can trigger a strong ERP.

Thanks to the differences in EEG recordings for correct and incorrect decisions illustrated in Fig. 4, it is possible to exploit them within a cBCI (typically in combination with other measurements) to estimate the probability of each decision being correct, which is a form of confidence.

#### Groups assisted by a collaborative BCI are more accurate than traditional groups

**Figure 5:**
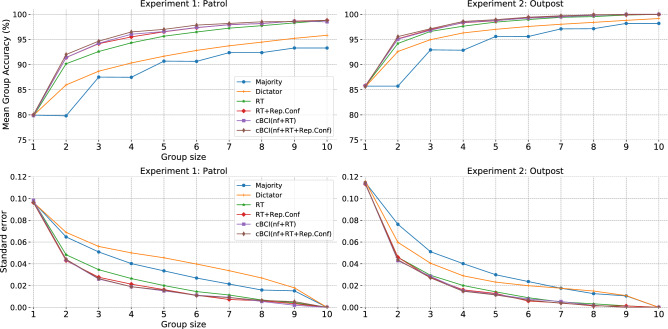
The average group accuracies of all possible groups of sizes one to ten formed from the ten participants for Experiments 1 and 2 and the corresponding standard error of the mean (computed using a boostrapping procedure). Results for the following decision aggregation strategies are shown: majority (in blue), dictatorial decisions (in orange), RT (in green), RT and reported confidence-based estimation (in red), a cBCI using neural features and RT (in purple), and a cBCI using neural features, RT and reported confidence (in maroon).

Figure 5 also shows the mean accuracies and standard errors of the mean for individuals and groups of sizes two to ten using different cBCI-based decision support systems for Experiments 1 (left) and 2 (right). The different cBCIs use different inputs: (a) neural features, RTs and reported confidence (cBCI(nf+RT+Rep.Conf) in maroon); (b) neural features and RTs (cBCI(nf+RT) in purple); and (c) reported confidence and RTs (RT+Rep.Conf in red). For reference we also report the results obtained from decision support systems that use standard majority (Majority in blue), only RTs (RT in green) and dictatorial system (Dictator in orange). To reconstruct the RT, we employed an algorithm (see Methods section) that performed pairwise comparisons of the frames preceding the response to identify the one where a significant difference occurred. The time where such a frame was presented is taken to be the stimulus onset. In the dictatorial system, the system identifies a group’s most skilled member based on individual accuracy and the other member of the group trusts the best member’s judgement to make the final decision.

We performed pairwise comparisons of the accuracies of all confidence estimation methods discussed above over all groups of sizes two to nine using two-tailed Wilcoxon signed rank test with Holm-Bonferonni adjustments (More information on the validation our statistical comparison approach is provided in the supplementary section of this article).

For Experiment 1, cBCI(nf+RT+Rep.Conf) is significantly better than Majority (test statistics $=0$, $p < 0.001$, $45 \ge$ Degree of Freedom $\le 252$, effect size $=0$), Dictator ($0<$ test statistics $< 53$, $p < 0.001$, $45 \ge$ Degree of Freedom $\le 250$, $0<$ effect size $< 4.2$), RT ($0<$ test statistics $\le 166.5$, $p < 0.001$, $39 \ge$ Degree of Freedom $\le 241$, $0<$ effect size $< 15.2$), RT+Rep.Conf ($53<$ test statistics ≤ 2376, $p < 0.001$, $35 \ge$ Degree of Freedom $\le 218$, $7.9<$ effect size $< 149.7$) and cBCI(nf+RT) ($29<$ test statistics $\le 2774$, $p < 0.001$, $35 \ge$ Degree of Freedom $\le 221$, $4.3<$ effect size $< 175$) for groups of size two to eight. In particular, this last comparison indicates the utility of having neural features extracted from EEG among the inputs to a decision support system. Similarly, for Experiment 2, cBCI(nf+RT+Rep.Conf) is significantly superior to Majority (test statistics $=0$, $p < 0.001$, $45 \ge$ Degree of Freedom $\le 250$, effect size $=0$), Dictator ($0<$ test statistics $< 1932$, $p < 0.001$, $44 \ge$ Degree of Freedom $\le 242$, $0<$ effect size $< 122$), RT ($0<$ test statistics $\le 1236$, $p < 0.003$, $21 \ge$ Degree of Freedom $\le 205$, $0<$ effect size $< 86$) and cBCI(nf+RT) ($20<$ test statistics $\le 3114$, $p < 0.002$, $19 \ge$ Degree of Freedom $\le 156$, $0<$ effect size $< 197$) for groups of size two to eight. It is also superior to RT+Rep.Conf ($52<$ test statistics $\le 1236$, $p < 0.003$, $21 \ge$ Degree of Freedom $\le 205$, $0<$ effect size $< 86$) for groups of size two, three, four, five and seven. The less marked superiority of cBCI(nf+RT+Rep.Conf) over RT+Rep.Conf in this experiment is a reflection of the weaker differences in the ERPs associated with correct and incorrect trials in Experiment 2 (see Fig. 4 (right))(Additional statistical comparisons between methods can be found in Table S1a–g and Table S2a–g within the supplementary section of this article.).

As one can see in Fig. 5, the differences in performance between all confidence-weighted methods (RT, RT+Rep.Conf and all cBCIs) and standard majority are larger for even-sized groups than for odd-sized groups. This is caused by the different behaviours exhibited by majority and the confidence-weighted methods in the presence of ties (which are only possible groups of even size). In the presence of a tie, standard majority breaks the tie by flipping a coin (there is no better strategy, since classes are equiprobable). On the contrary, with the confidence-weighted methods ties are simply resolved by picking the class with the higher total confidence, which is more often than not the correct decision. This is particularly beneficial with groups of size two, which present the biggest improvement over traditional methods because pairs are more likely to generate ties than larger groups, and hence they benefit the most from the ability of breaking ties in favour of correct decisions afforded by the weighted confidences derived from cBCIs, RTs and reported confidence.

#### Decision confidences derived from physiological and neural measures are good at assessing one’s decision

**Figure 6:**
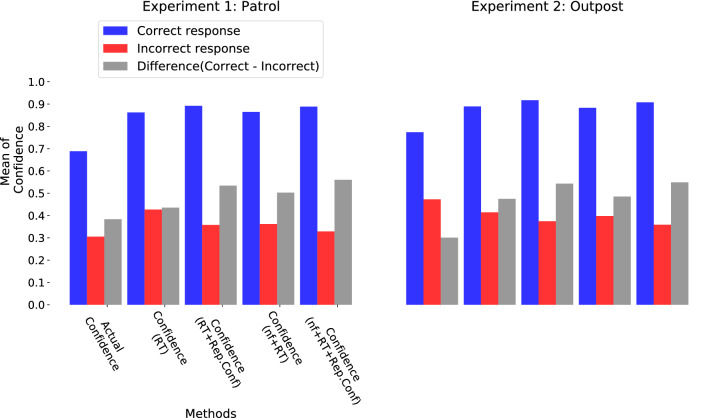
Distribution of the mean of actual confidence reported by the participants (Actual Confidence) and the estimated confidences derived from RT (confidence(RT)), RT and reported confidence (confidence(RT+Rep.Conf)), cBCI confidence based on neural features and RT (cBCI confidence(nf+RT)) and finally, cBCI confidence based on neural features, RT and reported confidence (cBCI confidence(nf+RT+Rep.Conf)) for correct (in blue) and incorrect decisions (in red) made by the participants in Experiment 1 (left column) and 2 (right column), respectively. The grey bar indicates the difference in mean of confidences (both actual and estimated) for correct and incorrect decisions.

Figure 6 presents the mean confidence available from decision support systems based on:(a) reported confidence, (b) RT only (confidence(RT)), (c) RT and reported confidence (confidence(RT+Rep.Conf)), (d) neural features and RT (cBCI confidence(nf+RT)), and (e) neural features, RT and reported confidence (cBCI confidence(nf+RT+Rep.Conf)). Results for the ten participants for Experiments 1 and 2 are shown in the bar charts on the left and right of the figure, respectively. The confidences are divided into two classes, associated with correct (in blue) and incorrect (in red) responses, respectively. The differences between these two conditions are also reported (in grey).

It is clear from the figure that participants reported higher confidence when they responded correctly than when they erred (Wilcoxon-signed rank test, $p < 0.007$, for both experiments). This is expected, as confidence is a self-assessment of one’s decisions and, therefore, decisions with high confidence should more likely be correct than incorrect.

The differences in average confidence for the incorrect and correct responses shown in the figure (grey bars) indicate that all decision support systems introduced in this paper have at least as good a separation between the two classes as the actual reported confidence. In fact, taken in the order shown in the figure, the separation is 5.22%, 15.06%, 11.95% and 17.66% better than the reported confidence in Experiment 1 and 17.38%, 24.22%, 18.43% and 24.80% better than the reported confidence in Experiment 2. While these differences are consistent, individually they are not statistically significant. However, the picture changes drastically when, later, we will use these decision support systems to aid group decision making. There we will not only see that the apparent superiority of all the decision support systems against the standard reported confidence is real, but we will also see that the cBCI based on the neural features, RT and reported confidence is also superior to all the other decision support systems.

#### Anytime morphing between decision support systems gives optimal time vs accuracy trade-offs

As noted from Fig. 5, the cBCI based group-decision making system with reported confidence (cBCI(nf+RT+Rep.Conf)) as an additional feature is superior in performance to the other alternatives. A limitation of group decision-making systems based on reported confidence is that a decision can only be made after the members in the group have registered their confidences. These processes can easily take several seconds, which may be incompatible with the decision times required by many real-world situations. The cBCI-based group decision-making system not using reported confidence can produce a less accurate decision sooner, that is immediately after all group members have provided a response. This may still require an excessively long time, especially in large groups. To get even quicker decisions, as we suggested in^71^, one could take a decision after the fastest *N* responders have cast their vote.

Here we explored an alternative strategy that tries to obtain the best compromise from accuracy and decision speed from all the above mentioned methods. The approach effectively smoothly morphs between the fastest system, where only the quickest responder determines the group decision, to the slowest one, where all participants have reported decisions and confidences and all contribute.

The strategy gathers all of the information (neural signals, decisions and reported confidence) available from any number of group members at any given time after the fastest responder has provided a decision. It then feeds such information to the appropriate types of decision support system. Such systems must all speak the same language; i.e. they must return an evaluation of the probability of the decision provided by a participant being correct (confidence). This makes it possible to form group decisions—via a confidence-weighted majority vote—even if the confidence of participants was evaluated by different systems. In this way, at any time a group decision is available. The decision is then updated as soon as new information is available, making such a system an anytime algorithm^80^.

**Figure 7:**
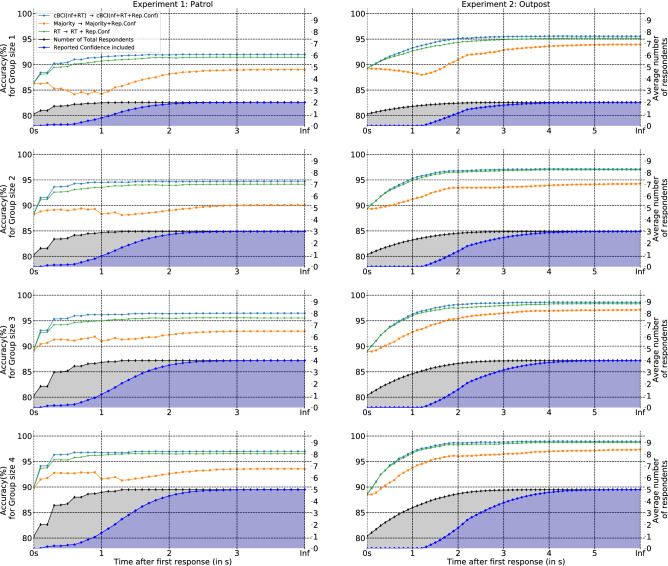
Average accuracies of Experiment 1: patrol experiment (left column) and Experiment 2: outpost experiment (right column) obtained using the following pairs of decision support systems: (**a**) two cBCIs based on neural features and RT (cBCI(nf+RT)), and neural features, RT and reported confidence(cBCI(nf+RT+Rep.Conf)) (line plot in blue), (**b**) a decision support system based on RT (RT) and a combination of RT and reported confidence (RT+Rep.Conf) (line plot in green), and finally (**c**) a decision support system made of standard (Majority) and confidence-weighted majority system (Majority+Rep.Conf) updated at every 100ms after the first response. The shaded region in black indicates the average number of total respondents (which is determined by the length of the secondary ordinate axis) at every 100ms time interval and the shaded region in blue indicates the average number of respondents who had reported their confidences.

We applied this morphing strategy to three pairs of decision support systems: (1) the two cBCIs tested in Fig. 5, (2) a decision support system based on RT and one based on RT as well as reported confidence, and (3) standard and confidence-weighted majority voting. For the standard majority system, confidence was a static quantity equal to the average accuracy of all participants in the training set. Figure 7 reports the results obtained with the corresponding anytime decision support systems.

More specifically, the figure shows how the accuracies of groups of size two to five and for Experiment 1 (right column) and Experiment 2 (left column) vary as a function of time after the first response for each of the three anytime systems. Decisions were updated by each system every 100 ms. The figure also shows how many members on average had responded by each time (shaded region with secondary ordinate axis) and the number of responders who had also reported their confidence (shaded blue region).

It is clear from the figure that both the cBCI and the system based on RTs present a monotonically increasing accuracy profile, when the more time available for the group decision, the more accurate that decision. Interestingly, in most cases, after a rather rapid transient, accuracy tends to plateau, which suggests that near optimal decisions can be obtained well before all participants have responded and reported their confidence. It is also clear that, thanks to the use of neural information, the cBCI always has an edge over the purely behavioural system based on RT. The cBCI anytime method also outperforms the majority-based system.

Somehow surprisingly, the accuracy of the majority-based group-decision system is not always a monotonic function of time. This effect is associated with the fact that the best performers in a group are often also the fastest responders. In the majority system all responses have the same weight, until confidence values are available. During this period, as more and more weaker members cast their vote, the group accuracy may fail to increase (or, worse, it can even decrease) over time. The situation improves as more and more members express their confidence. However, accuracy eventually plateaus to a markedly lower value than for the other systems.

### Discussion

Metacognitive processes make decision-makers consciously or unconsciously aware of the likelihood of their decision being correct, through a feeling that we call confidence. In our previous research^33,71,75,81,82^, we found that, when decision makers act in isolation, i.e. in the absence of communication or peer pressure, a BCI can provide estimates of confidence on a decision-by-decision basis that are often more correlated with decision correctness than the confidence reported by participants themselves. We then used these estimates to improve the performance of groups of decision-makers by simply weighing decisions by the corresponding BCI confidence–a system that we call a collaborative BCI, or cBCI for short. All of our tests to date involved decisions based on either static images or speech.

In this paper, we have extended and then applied our cBCI to assist with perceptual decisions in two dynamic realistic environments. In the first environment, participants viewed video feeds showing the perspective of a user walking along a dark corridor and trying to identify possible threats. The second environment simulated an even more realistic situation: an outpost at night where potential threats would quickly walk towards the outpost and where the outcome of an erroneous and/or slow decision could be very severe. In both these situations one could imagine that an automated computer–vision system for target detection could be a better solution. However, for ethical reasons, many decisions in the military domain that can lead to possible fatalities (including those represented in the two scenarios studied in this paper) cannot be made by an AI system in full autonomy. A human needs to be always in the loop^83,84^. For this reason, it makes sense to augment and assist human-decision making using AI-based technologies.

In addition to dealing with the challenges imposed by such environments, we decided to address an additional challenge: in many real-world applications precise RTs are unavailable because situations requiring a decision present themselves at random times and users must realise by themselves that a situation requires a decision in the first place. For the first time, our decision-support systems are capable of reconstructing RTs, thereby dealing with this challenge and making them even more applicable in practice.

Despite these challenges, for both environments, results confirm that the cBCI based on neural features, RT and reported confidence is significantly better than traditional standard majority and dictatorial system and also, most often, other machine-learning-based decision-support systems relying on behavioural data (RT and reported confidence) to estimate confidences. The RT based decision-support systems are also significantly better than standard majority and dictatorial system. So, in the absence of neural system infrastructure such as an EEG system, the RT based systems can be implemented as an alternative albeit with a small compromise in performance.

Group decision support systems that rely on reported confidence present the drawback that decisions can only be made after the process of assessing and reporting individual confidence values is complete, which may take an additional few seconds. Our cBCI based on neural features and just RT does not present this problem and is the second-best choice, being significantly better than both majority and also the decision-support system relying on RT to estimate confidences.

It is clear from our results that using reported confidence as an additional feature allows our decision support systems to provide more reliable estimates of the probability of correctness. While, as noted above, confidence reporting requires extra time, it is often the case that by the time the slowest responders in a group have provided their decisions (thereby enabling the group decision), the fastest ones have also reported their confidence. Also, there may be cases where one can afford more time for the decision, which would allow more group members to report their confidence.

With this in mind, in this paper we proposed and tested three *anytime* decision support systems (both behavioural and cBCI-based). Our anytime systems estimate the decision confidence for all available responders in the group at any given time (after the first response) using a decision support system trained to work *without* the reported confidence as an input for all users who did not have time to report the confidence and one trained to work *with* the reported confidence for all users who reported it. It then makes the group decision. This decision, however, may change over time as more and more users make decisions and report their confidence.

Results indicate that the anytime cBCI-based decision support system is superior to the two behavioural anytime systems in the test environments considered. They also suggest that after a certain experiment-dependent time, group accuracy does not further improve significantly with time. So, our systems are on par in terms of accuracy with corresponding non-anytime versions, but are faster. If an application requires even faster decisions, our anytime systems can provide such decisions, but at the cost of a reduced group accuracy. For these reasons, such systems are may be particularly suitable for perceptual decision making scenarios in defence, policy-making and healthcare, where rapid decision-making may be needed.

Although our two environments have been designed to mimic realistic situations, they are still crude approximations of the rich set of sensory inputs and bodily reactions that people might encounter in real-world situations, particularly in the presence of real (as opposed to simulated) risk. Also, our participants were tested in very controlled lab conditions (e.g., they sat in a comfortable chair; there was very little noise and other distractions from the environment; the experiments were of a limited duration, thereby only inducing mild fatigue; etc.). While these conditions are not completely atypical of the military domain (e.g., in the virtual cockpit of a drone, or in remote/distributed C2 decision-making), there are many real, complex environments where they do not apply. In such cases, one should expect that, in general, poorer results might be obtained. Muscular artefacts produced by physical activity may not necessarily be an issue, as we recently reported in^85^, where we found that walking on a treadmill did not produce any negative effect on individual performance in the patrol task, instead improving cBCI/group performance, likely due to increased level of alertness associated with walking. However, EEG signals would drastically be affected by strenuous exercise, intense accelerations, intense mental fatigue, etc., which would likely render the cBCI approach presented here inapplicable.

Another limitation of the approach is that cBCIs have mostly been tested in assisting perceptual decision making in situations where there are only two options and there is some form of time pressure and/or where the perceptual information is available only for a short time, is inconsistent or overwhelmingly detailed. Only a fraction of all situations have these characteristics, many involving strategic decisions where resources (rather than time) are limited and where^5^ more than two choices are available to make a decision. We are currently exploring these situations through a joint US DoD/UK MoD research initiative (https://basicresearch.defense.gov/Pilots/BARI-Bilateral-Academic-Research-Initiative/). There, we are extending our cBCI application to more complex problems in which, for example, decisions do not necessarily have a correct or incorrect choice, and are not just based on perceptual input given in the current trial, but also on information gained and decisions made in past trials. In this case, additional factors need to be taken in consideration, such as, for example, problem framing^5^, which can change decisions as individual perspectives change, and the notion that humans decisions often bring to solution that are not necessarily optimal, but, rather, satisfactory, as suggested in bounded rationality^86^.

### Methods

#### Participants

Two different groups of ten healthy participants took part in the experiments mentioned above: six females, four left-handed, age = $35.4\pm 2.6$ years in Experiment 1, and four females, one left-handed, $\hbox {age} = 34.3\pm 11.67$ years in Experiment 2. All the participants self-reported to have normal or corrected-to-normal vision and no history of epilepsy. All participants were provided with a participant information sheet informing them about the nature and objective of the experiment and they were also briefed about the experiments before the start of the session. Then they signed an informed consent form if they agreed to move ahead with the experiment. The participants were comfortably seated in a medical chair at about 80 cm from an LCD screen. After the experiment, the participants received a monetary remuneration for their time of £16 in Experiment 1 and £12 for their participation plus an additional remuneration of up to £6 (depending on their performance) in Experiment 2. The total duration of the experiments was around 50 to 70 minutes depending on the speed of response of the participants.

#### Stimuli description

*Experiment 1: Patrol* Participants were presented with video sequences (frame rate = 4 Hz) of a dynamic environment representing the viewpoint of a user walking at a constant pace along a corridor, where characters could appear from doorways, located on either side of the corridor, for one frame (Fig. 2(b)). Each participant had to decide, as quickly as possible and within 2.5s, whether the character crossing the corridor was wearing a helmet (by clicking the left mouse button) or a cap (by clicking the right mouse button). After reporting their decision, participants were asked to indicate, within 2 s and using the mouse wheel, their degree of confidence in that decision, using an 11-point scale (from 0=not confident, to 100=very confident, in steps of ten). The experiment was composed of 12 blocks of 42 trials, each trial corresponding to a doorway encountered while walking down the corridor. In each block, 14 trials had empty doors (no decisions required), 14 trials contained a person wearing a helmet, and 14 trials contained a person wearing a cap. The sequence of trials was randomised, and the same sequence was used with all participants, which allowed the simulating of group decisions offline. Prior to the start of the experimental session, each participant underwent a brief training session of 21 trials (approximately two minutes) to familiarise them with the task.

*Experiment 2: Outpost* In this experiment, each participant viewed a scene simulating their being at an outpost and viewing an area with a house and several trees through a (simulated) night vision camera (Fig. 2(c)). In each trial, a character appeared from a distance, either from the house or from the adjoining forest cover on either side and walked towards the outpost. The video sequence had a frame rate of 10 Hz. The participant had to decide, as quickly as possible, whether the character was wearing a helmet (by clicking the left mouse button) or a cap (by clicking the right mouse button). After each response, participants were asked to indicate (within 2 s) their decision confidence on a scale from 0 (not confident) to 100 (very confident) in steps of ten by using the mouse wheel. The experiment included a point-based reward system considering the correctness of the decision and the RT of the participant. When a participant made a correct decision, they gained more points for faster RTs than for slower ones. In the case of incorrect responses, points were deducted (penalty) proportionally to the RT. Moreover, to simulate the risk in waiting for too long to make a decision, in each trial the character disappeared after a random time. If the participant did not make any decision by then, the trial was labelled as *incorrect* and a maximum penalty was applied. At the end of the experiment, the number of points accumulated by the participant was converted into currency (between £0 and £6) to determine the extra remuneration for the volunteer. The point-based reward system attempted to simulate a high-pressure critical decision-making situation where the user must respond correctly and as quickly as possible. The experiment was composed of six blocks of 60 trials. In each block, 30 trials contained a person wearing a helmet, and 30 trials contained a person wearing a cap. The sequence of trials was randomised, and the same sequence was used with all participants to enable the simulating of group decisions offline. Prior to the start of the experimental session, each participant underwent a brief training session of 15 trials (approximately two minutes) to familiarise them with the task.

#### Data recording and pre-processing

A Biosemi ActiveTwo EEG system was used to record the neural signals from 64 electrode sites following the 10-20 international system. The EEG data were sampled at 2048 Hz, referenced to the mean of the electrodes placed on the earlobes, and band-pass filtered between 0.15 to 40 Hz to reduce electrical noise. Artefacts caused by eye-blinks and other ocular movements were removed using a standard subtraction algorithm based on correlations to the averages of the differences between channels Fp1-F1 and Fp2-F2. EEG signals, RT, reported confidence, skin conductance, heart rate variability, respiration frequency and profile, pupil dilation, eye movements and eye blinks were simultaneously recorded during the experiments. RTs were measured by time-stamping the clicks of an ordinary USB mouse when the participant had responded. For this study, we used only the EEG, RTs and the reported confidence.

For each trial, the EEG data were segmented into response-locked epochs, starting from 1700 milliseconds (ms) before the response and lasting for 1900 ms. The epochs were then detrended and low-pass filtered at a pass band of 0–14 Hz and a stop band of 16-1024 Hz with an optimal Finite Impulse Response (FIR) filter designed with the Remez exchange algorithm. Finally, the data were down-sampled to 32 Hz and each epoch was trimmed by removing 200 ms from the beginning and end of the epoch. The remaining 1500 ms of the epochs were further analysed.

#### Reconstruction of response time

**Figure 8:**
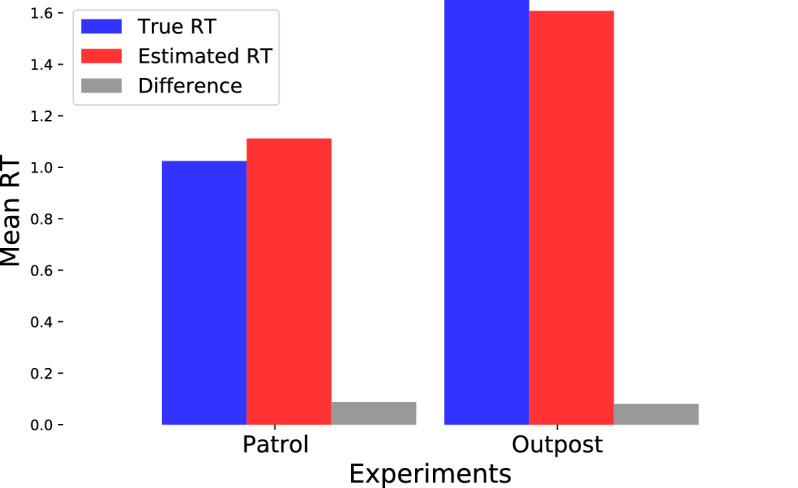
Comparison of the average of true RT (in blue) and estimated RT (in red) across 10 participants for Patrol and Outpost experiment.

In a real-life situation, while it can be very clear when an individual reacts to an event, it is not always necessarily clear when that event has occurred. In our study, we simulated exactly this kind of circumstance, where the reaction (a button press in our experiment) of the participant was known to the BCI system, but information on what caused it and when was not known. Hence, to reconstruct the RT for such situations, we needed to detect the onset of stimuli (*‘stimuli detector’*). To achieve this, in each trial we parsed back each frame from the time of the response (‘response event’) until a frame was found where the change in average RGB values with respect to the preceding frame was above a certain threshold, which was considered to represent the moment of appearance of the character (‘stimulus event’) that eventually caused the button press. Then, the reconstructed RTs were calculated by subtracting the stimulus event time from their corresponding response event time. Figure 8 shows the difference between the average of the true (in blue) and estimated (in red) RT across all participants in the patrol and outpost experiment. The efficacy of our RT reconstruction algorithm is evident by the small absolute difference between the true and estimated RT for both the experiments (0.0875s in patrol experiment and 0.0807s in outpost experiment). In the patrol experiment, the estimated RT is larger than the true RT because the stimuli detector sometimes missed the characters on-screen, in which case the stimulus onset is taken to be the onset of the previous stimulus. On the contrary, the estimated RT is lower than the true RT in the outpost experiment because in some circumstances, the stimuli detector system identified the character on-screen later than the actual onset. Nevertheless, the small difference in the RTs yielded no significant changes in the confidence estimation of the decision-support methods.

#### Labelling the epochs

Our cBCI approach to group decision-making assigns higher weights to individual decisions where a participant was confident (and more likely to be correct) and lower weights to decisions where the participant was unsure (and more likely to be incorrect)^13,82^. To attain this, we trained our cBCI system using the *correctness* of individual decisions, which is available to the cBCI in the training set. The trials in which the participant made a correct decision were labelled as *correct* while those where the participant made an incorrect decision were labelled as *incorrect*. In this approach, the cBCI is trained to predict whether the user made a correct or an incorrect decision rather than decoding targets and non-targets. The same approach was used to train decision support systems only employing behavioural data (RT and reported confidence) to make their predictions.

#### Estimation of individual decision confidences

Common Spatial Pattern (CSP)^87^ was used to extract characteristic neural features from each epoch that can distinguish between the *correct* and *incorrect* labelled trials. The main idea behind CSP is to transform the multi-channel EEG data into a low-dimensional spatial subspace using a projection matrix, that can maximise the variance of two-class signal matrices. In our study, we have used an eight-fold cross validation to split the data into training and test sets. Each training set is used to compute a CSP projection matrix, which is then applied to transform the data into a low-dimensional subspace for the corresponding test. The variances for the two classes (i.e., correct and incorrect responses) are largest in the first and the last dimensions of the subspace. So, the logarithm of the variances of the first and the last spatial subspaces along with the reconstructed RT (which is known to influence decisions^88^) and reported confidence (when required) were used as features for a random forest model to predict the decision confidence. The model was fitted using 100 decision trees and *Gini* criterion. The random forest approach fits sub-samples (with replacement) of the dataset on various individual decision trees and the final output is an average of the results obtained from each one. This form of estimation improves the prediction accuracy and controls over-fitting. Thanks to cross validation all confidence estimates were obtained from test sets, i.e., they were obtained from inputs not previously seen by the machine learning model. This method was adopted to avoid over-fitting and deliver robust confidence estimates even in the presence of small data samples. A similar random forest model was used to calibrate the decision confidence of trials from their corresponding response time (when required).

#### Formation of groups

Formally, each participant, *p*, has a final confidence weight $w_{p,i}(t)$ for each trial *i*, obtained either from their decision confidence (cBCI or not) with or without reported confidence, depending on the time *t* after the stimulus event. Group decisions are then made as follows:

1 $$\begin{aligned} d_{group,i}(t) = \texttt {sign}\left( \sum _{p=1}^{m}w_{p,i}(t)\cdot d_{p,i}(t)\right) , \end{aligned}$$

where $d_{p,i}(t)$ is the decision of participant *p* in trial *i* when checked at time *t*. Both $w_{p,i}(t)$ and $d_{p,i}(t)$ are assumed to be 0 if the participant has not yet made a decision at time *t*.

Groups of size $m{=}2,\dots ,10$ were formed offline by considering the $$\left( {\begin{array}{c}10\\ m\end{array}}\right)$$ combinations of the 10 participants. Hence, there are

- 45 groups of size two (for example (1, 2), (2, 3), (9, 10) and more),
- 120 groups of size three (for example (1, 2, 3), (1, 3, 4), (2, 3, 4) and more),
- 210 groups of size four (for example (1, 2, 3, 4), (1, 3, 4, 5), (2, 3, 4, 7) and more),
- 252 groups of size five (for example (1, 2, 3, 4, 5), (1, 3, 4, 5, 9), (2, 3, 4, 7, 8) and more),
- 210 groups of size six (for example (1, 2, 3, 4, 5, 6), (1, 3, 4, 5, 7, 9), (2, 3, 4, 5, 7, 8) and more),
- 120 groups of size seven (for example (1, 2, 3, 4, 5, 6, 7), (1, 3, 4, 5, 7, 9, 10), (2, 3, 4, 5, 6, 7, 8) and more),
- 45 groups of size eight (for example (1, 2, 3, 4, 5, 6, 7, 8), (1, 3, 4, 5, 7, 8, 9, 10) and more),
- 10 groups of size nine (for example (1, 2, 3, 4, 5, 6, 7, 8, 9), (1, 3, 4, 5, 6, 7, 8, 9, 10) and more), and
- 1 group of ten (for example (1, 2, 3, 4, 5, 6, 7, 8, 9, 10)).

#### Designing the anytime morphing approach to make group decisions

The *anytime* morphing approach works as follows: In a group of responders, when the first responder reacts to a stimulus event in the video feed by clicking a mouse button to signify the presence of a target or a non-target, a clock starts. Within a few milliseconds the software identifies the stimulus event and it can, therefore, reconstruct the RT for the first responder. The EEG data are also already available, and so a first approximation of confidence can be immediately computed by the BCI. The group decision at this stage is the decision of the first responder. Then, every 100 ms from the first response, the system looks for other members in the group who have responded, uses the first responder stimulus event to estimate their RTs, then computes their cBCI confidence and uses a corresponding weighted majority (Eq (1)) to produce the group decision (which may, therefore, change over time as more and more team members react to the stimulus). At every clock tick, the system also checks whether any of the team members who previously responded have also manually provided a confidence value. For those where this has happened, the reported confidence is added as input features to obtain a new cBCI-estimated confidence. Every time either the pool of responders changes or those who have expressed a confidence changes, the decision weights and, then, the group decision are updated, until all group members have made their decisions and reported their confidence.

**Figure 9:**
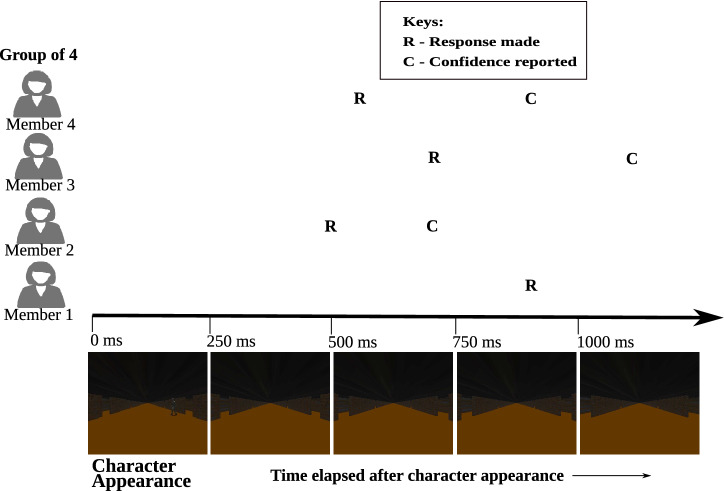
An example of the *anytime* cBCI system for group of size four (see explanation in the main text).

An illustration on the workings of the *anytime* cBCI system (and the other two behavioural anytime decision support systems tested in the paper) is shown in Fig. 9. The polling of group members begins when the system detects the first response after the stimulus. At the first response made by Member 2, only the neural and reconstructed RT features are available to the system and, hence, the decision confidence is determined by the BCI. Some time after the first response, a second responder (Member 4) joins the first but the reported confidence is not yet available for both responders. Hence, up until this moment, the BCI uses only the neural and reconstructed RT features to decide the decision confidence for both participants like in our normal (non-anytime) cBCI. The situation does not change until, 700 ms after the first response, the reported confidence of the first responder is available and, hence, it is added as a new feature to the existing BCI to determine a new decision confidence for Member 1, and a third participant (Member 3) has provided a response. So, if a situation demands to report a decision at around 700 ms after the onset of stimuli, then based on our example, the *anytime* BCI will make the decision based on neural and reconstructed RT features for two responders (Members 3 and 4) and the neural, RT and reported confidence features for one responder (Member 1). Obviously, eventually also the fourth group member (Member 1) expressed an opinion and given enough time would also provide a reported confidence (not shown in the figure), after which the group decision would be final.

## Supplementary information

Supplementary material 1 (pdf 523 KB)

## Data Availability

The data that support the findings of this study are available on request from the corresponding author [S.B. and R.P.]. The data are not publicly available due to funding restrictions.

## Code availability

The code that support the findings of this study are available on request from the corresponding author [S.B. and R.P.]. The data are not publicly available due to funding restrictions.
