# Supplementary material for: Anytime collaborative brain–computer interfaces for enhancing perceptual group decision-making
Source: Sci Rep. 2021 Aug 20;11:17008. doi: 10.1038/s41598-021-96434-0 (PMC8379268; doi:10.1038/s41598-021-96434-0)
Supplement: Supplementary file 1 — Supplementary material 1 (pdf 523 KB) [file 41598_2021_96434_MOESM1_ESM.pdf]

Supplementary Information

Anytime Collaborative Brain-Computer Interfaces for Enhancing Perceptual Group Decision-Making

Saugat Bhattacharyya[1,3,*], Davide Valeriani[2], Caterina Cinel[1], Luca Citi[1], and Riccardo Poli[1,*]

[1]Brain Computer Interfaces and Neural Engineering Laboratory

School of Computer Science and Electronic Engineering

University of Essex, Wivenhoe Park, Colchester, CO4 3SQ, UK.

[2]Department of Otolaryngology, Head and Neck Surgery

Massachusetts Eye and Ear, Harvard Medical School, 243 Charles St, Boston MA, USA

[3]School of Computing, Engineering & Intelligent Systems

Ulster University, Northland Road, Londonderry, BT48 7JL, UK

[*]s.bhattacharyya@ulster.ac.uk, rpoli@essex.ac.uk

Supplementary Information 1: Pairwise comparison of group accuracies

For each group size and pair of decision integration methods, we compared the accuracy obtained by all possible groups with both methods. We then applied the Wilcoxon signed rank test to obtain a measure of statistical significance. As correctly noted by one of the reviewers, the Wilcoxon signed rank test requires that all observations are independent from each other: this assumption is potentially violated in our case, as different groups may share a significant proportion of individuals. To verify if this could be an issue, we have put ourselves in the null hypothesis that the pairs of aggregation methods being tested do not make a difference to the recorded group performance. So, we went through each group and randomly swapped the accuracy obtained by the two methods under test and then run the Wilcoxon signed rank test. We repeated this process 10,000 and identified the 5-th percentile in the resulting dataset. This represents the standard confidence level, α=0.05. The p-values returned by the Wilcoxon test at the 5-th percentile for all group sizes and pairs of aggregation methods were extremely close to 0.05. They were 0.05041 $\pm$ 0.00205 for the patrol experiment, and 0.05021 $\pm$ 0.00441 for the outpost experiment. This indicates that any potential issues related to the independence of samples are minimal and validated the use of the Wilcoxon signed rank test in our experiments.

Figure 5 in the main file shows the mean accuracies for individuals and groups of sizes two to ten using different cBCI-based decision support systems for Experiments 1 and 2 . The different systems use different inputs: standard majority (Majority in blue), dictatorial system (Dictator in orange), RTs (RT in green), RT and reported confidence-based estimation (RT+Rep.Conf in red), a cBCI using neural features and RT (cBCI(nf+RT) in purple), and a cBCI using neural features, RT and reported confidence (cBCI(nf+RT+Rep.Conf) in maroon).The following tables shows the statistical pairwise comparison of each decision system using two-tailed Wilcoxon signed rank test with Holm-Bonferonni adjustments for experiment 1 (Supplementary Table S1a-S1f) and 2 (Supplementary Table S2a-S2f).

Table S1a. Statistical results of pairwise comparisons between different decision support system using two-tailed Wilcoxon signed rank test with Holm-Bonferonni adjustments for experiment 1 and group size 2

**Method1**	**Method2**	**Test statistics**	**Corrected P-value**	**DoF**	**Effect size**
Majority	Dictator	0.0	7.736e-08	45.0	0.0
Majority	RT	0.0	7.736e-08	45.0	0.0
Majority	RT+conf	0.0	7.736e-08	45.0	0.0
Majority	nf+RT	0.0	7.736e-08	45.0	0.0
Majority	nf+RT+conf	0.0	7.736e-08	45.0	0.0
Dictator	RT	18.0	1.556e-07	44.0	2.6832
Dictator	RT+conf	0.0	7.736e-08	45.0	0.0
Dictator	nf+RT	3.0	7.736e-08	45.0	0.4472
Dictator	nf+RT+conf	0.0	7.736e-08	45.0	0.0
RT	RT+conf	20.0	2.242e-07	43.0	2.9814
RT	nf+RT	26.5	2.242e-07	44.0	3.9504
RT	nf+RT+conf	4.0	7.736	45.0	0.5963
RT+conf	nf+RT	458.0	0.8562	43.0	68.275
RT+conf	nf+RT+conf	157.0	0.00015	44.0	23.404
nf+RT	nf+RT+conf	114.5	0.00012	41.0	17.069

Table S1b. Statistical results of pairwise comparisons between different decision support system using two-tailed Wilcoxon signed rank test with Holm-Bonferonni adjustments for experiment 1 and group size 3

Method1	Method2	Test statistics	Corrected P-value	DoF	Effect size
Majority	Dictator	2232.0	0.00276	116.0	203.753
Majority	RT	0.0	2.948e-20	120.0	0.0
Majority	RT+conf	0.0	2.948e-20	120.0	0.0
Majority	nf+RT	0.0	2.948e-20	120.0	0.0
Majority	nf+RT+conf	0.0	2.948e-20	120.0	0.0
Dictator	RT	397.0	1.256e-16	120.0	36.241
Dictator	RT+conf	103.0	4.505e-19	118.0	9.4025
Dictator	nf+RT	86.0	4.878e-19	116.0	7.851
Dictator	nf+RT+conf	45.0	1.184e-19	118.0	4.108
RT	RT+conf	56.5	4.505e-19	115.0	5.158
RT	nf+RT	4.5	9.996e-20	116.0	0.411
RT	nf+RT+conf	0.0	3.143e-20	119.0	0.0
RT+conf	nf+RT	2285.5	0.1121	105.0	208.637
RT+conf	nf+RT+conf	446.0	6.800e-12	98.0	40.714
nf+RT	nf+RT+conf	315.5	4.696e-13	97.0	28.801

Table S1c. Statistical results of pairwise comparisons between different decision support system using two-tailed Wilcoxon signed rank test with Holm-Bonferonni adjustments for experiment 1 and group size 4

Method1	Method2	Test statistics	Corrected P-value	DoF	Effect size
Majority	Dictator	2472.5	1.334e-21	208.0	170.618
Majority	RT	0.0	4.929e-35	210.0	0.0
Majority	RT+conf	0.0	4.929e-35	210.0	0.0
Majority	nf+RT	0.0	4.929e-35	210.0	0.0
Majority	nf+RT+conf	0.0	4.929e-35	210.0	0.0
Dictator	RT	1000.5	2.535e-28	203.0	69.041
Dictator	RT+conf	198.0	2.684e-33	205.0	13.663
Dictator	nf+RT	82.0	2.154e-34	208.0	5.658
Dictator	nf+RT+conf	1.0	2.154e-34	205.0	0.069
RT	RT+conf	472.0	1.564e-29	193.0	32.571
RT	nf+RT	34.0	2.154e-34	206.0	2.346
RT	nf+RT+conf	0.0	7.632e-35	208.0	0.0
RT+conf	nf+RT	2397.0	2.854e-17	184.0	165.409
RT+conf	nf+RT+conf	508.5	7.772e-29	190.0	35.090
nf+RT	nf+RT+conf	2352.5	2.654e-18	189.0	162.338

Table S1d. Statistical results of pairwise comparisons between different decision support system using two-tailed Wilcoxon signed rank test with Holm-Bonferonni adjustments for experiment 1 and group size 5

Method1	Method2	Test statistics	Corrected P-value	DoF	Effect size
Majority	Dictator	9633.0	2.960e-06	244.0	606.821
Majority	RT	0.0	6.468e-42	252.0	0.0
Majority	RT+conf	0.0	6.468e-42	252.0	0.0
Majority	nf+RT	0.0	6.468e-42	252.0	0.0
Majority	nf+RT+conf	0.0	6.468e-42	252.0	0.0
Dictator	RT	1027.0	1.256e-35	244.0	64.695
Dictator	RT+conf	178.0	9.877e-41	249.0	11.213
Dictator	nf+RT	232.0	9.738e-41	251.0	14.615
Dictator	nf+RT+conf	53.0	1.825e-41	250.0	3.339
RT	RT+conf	1137.5	1.143e-31	225.0	71.656
RT	nf+RT	1209.0	3.689e-32	230.0	76.160
RT	nf+RT+conf	80.0	5.827e-40	241.0	5.040
RT+conf	nf+RT	10315.5	0.6869	206.0	649.815
RT+conf	nf+RT+conf	2376.0	4.212e-24	218.0	149.674
nf+RT	nf+RT+conf	2774.0	5.555e-23	221.0	174.745

Table S1e. Statistical results of pairwise comparisons between different decision support system using two-tailed Wilcoxon signed rank test with Holm-Bonferonni adjustments for experiment 1 and group size 6

Method1	Method2	Test statistics	Corrected P-value	DoF	Effect size
Majority	Dictator	3372.5	7.728e-18	209.0	232.725
Majority	RT	0.0	4.914e-35	210.0	0.0
Majority	RT+conf	0.0	4.914e-35	210.0	0.0
Majority	nf+RT	0.0	4.914e-35	210.0	0.0
Majority	nf+RT+conf	0.0	4.914e-35	210.0	0.0
Dictator	RT	258.0	4.446e-32	200.0	17.804
Dictator	RT+conf	19.0	7.704e-35	208.0	1.311
Dictator	nf+RT	2.0	6.753e-35	208.0	0.138
Dictator	nf+RT+conf	0.0	4.914e-35	209.0	0.0
RT	RT+conf	486.0	2.355e-29	192.0	33.537
RT	nf+RT	137.0	2.167e-30	185.0	9.454
RT	nf+RT+conf	11.0	1.98e-34	205.0	0.759
RT+conf	nf+RT	7393.0	0.4047	178.0	510.165
RT+conf	nf+RT+conf	859.5	4.3658e-26	186.0	59.311
nf+RT	nf+RT+conf	526.5	2.106e-27	182.0	36.332

Table S1f. Statistical results of pairwise comparisons between different decision support system using two-tailed Wilcoxon signed rank test with Holm-Bonferonni adjustments for experiment 1 and group size 7

Method1	Method2	Test statistics	Corrected P-value	DoF	Effect size
Majority	Dictator	1428.0	4.075e-07	115.0	130.358
Majority	RT	0.0	2.584e-20	120.0	0.0
Majority	RT+conf	0.0	2.584e-20	120.0	0.0
Majority	nf+RT	0.0	2.584e-20	120.0	0.0
Majority	nf+RT+conf	0.0	2.584e-20	120.0	0.0
Dictator	RT	35.0	7.773e-20	118.0	3.195
Dictator	RT+conf	0.0	2.584e-20	120.0	0.0
Dictator	nf+RT	0.0	2.584e-20	120.0	0.0
Dictator	nf+RT+conf	0.0	2.584e-20	120.0	0.0
RT	RT+conf	297.5	9.116e-14	100.0	27.158
RT	nf+RT	169.5	1.653e-16	107.0	15.473
RT	nf+RT+conf	166.5	3.323e-17	111.0	15.199
RT+conf	nf+RT	2183.5	0.85302	94.0	199.325
RT+conf	nf+RT+conf	1272.5	1.956e-05	101.0	116.163
nf+RT	nf+RT+conf	1018.0	2.957e-06	97.0	92.930

Table S1g. Statistical results of pairwise comparisons between different decision support system using two-tailed Wilcoxon signed rank test with Holm-Bonferonni adjustments for experiment 1 and group size 8

Method1	Method2	Test statistics	Corrected P-value	DoF	Effect size
Majority	Dictator	81.5	5.131e-06	45.0	12.149
Majority	RT	0.0	7.026e-08	45.0	0.0
Majority	RT+conf	0.0	7.026e-08	45.0	0.0
Majority	nf+RT	0.0	7.026e-08	45.0	0.0
Majority	nf+RT+conf	0.0	7.026e-08	45.0	0.0
Dictator	RT	0.0	7.026e-08	45.0	0.0
Dictator	RT+conf	0.0	7.026e-08	45.0	0.0
Dictator	nf+RT	0.0	7.026e-08	45.0	0.0
Dictator	nf+RT+conf	0.0	7.026e-08	45.0	0.0
RT	RT+conf	74.5	0.00016	35.0	11.106
RT	nf+RT	92.5	4.518e-05	41.0	13.789
RT	nf+RT+conf	0.0	3.564e-07	39.0	0.0
RT+conf	nf+RT	222.5	0.61302	31.0	33.168
RT+conf	nf+RT+conf	53.0	4.900e-05	35.0	7.901
nf+RT	nf+RT+conf	29.0	1.174e-05	35.0	4.323

Table S2a. Statistical results of pairwise comparisons between different decision support system using two-tailed Wilcoxon signed rank test with Holm-Bonferonni adjustments for experiment 2 and group size 2

Method1	Method2	Test statistics	Corrected P-value	DoF	Effect size
Majority	Dictator	0.0	7.745e-08	45.0	0.0
Majority	RT	0.0	7.745e-08	45.0	0.0
Majority	RT+conf	0.0	7.745e-08	45.0	0.0
Majority	nf+RT	0.0	7.745e-08	45.0	0.0
Majority	nf+RT+conf	0.0	7.745e-08	45.0	0.0
Dictator	RT	178.5	0.0019	42.0	26.609
Dictator	RT+conf	60.0	1.922e-06	45.0	8.944
Dictator	nf+RT	74.0	5.366e-06	44.0	11.031
Dictator	nf+RT+conf	28.0	5.014e-07	44.0	4.174
RT	RT+conf	51.5	2.487e-06	43.0	7.677
RT	nf+RT	79.5	1.007e-05	43.0	11.851
RT	nf+RT+conf	28.5	7.157e-07	43.0	4.248
RT+conf	nf+RT	400.5	0.3813	43.0	59.703
RT+conf	nf+RT+conf	177.5	0.0021	41.0	26.460
nf+RT	nf+RT+conf	97.5	6.333e-05	41.0	14.534

Table S2b. Statistical results of pairwise comparisons between different decision support system using two-tailed Wilcoxon signed rank test with Holm-Bonferonni adjustments for experiment 2 and group size 3

Method1	Method2	Test statistics	Corrected P-value	DoF	Effect size
Majority	Dictator	1219.5	1.647e-07	111.0	111.325
Majority	RT	0.0	5.023e-20	118.0	0.0
Majority	RT+conf	0.0	2.935e-20	120.0	0.0
Majority	nf+RT	0.0	2.935e-20	120.0	0.0
Majority	nf+RT+conf	0.0	2.935e-20	120.0	0.0
Dictator	RT	1256.5	2.762e-08	116.0	114.702
Dictator	RT+conf	909.5	7.741e-11	116.0	83.026
Dictator	nf+RT	1128.0	8.382e-10	119.0	102.972
Dictator	nf+RT+conf	732.0	1.103e-11	113.0	66.822
RT	RT+conf	1443.5	0.00054	100.0	131.773
RT	nf+RT	1304.0	0.00054	96.0	119.038
RT	nf+RT+conf	939.0	3.827e-09	109.0	85.718
RT+conf	nf+RT	2321.0	0.483	100.0	211.877
RT+conf	nf+RT+conf	811.5	2.506e-05	86.0	74.079
nf+RT	nf+RT+conf	1089.0	2.506e-05	96.0	99.412

Table S2c. Statistical results of pairwise comparisons between different decision support system using two-tailed Wilcoxon signed rank test with Holm-Bonferonni adjustments for experiment 2 and group size 4

**Method1**	**Method2**	**Test statistics**	**Corrected P-value**	**DoF**	**Effect size**
Majority	Dictator	977.0	1.371e-28	206.0	67.419
Majority	RT	0.0	4.928e-35	210.0	0.0
Majority	RT+conf	0.0	4.928e-35	210.0	0.0
Majority	nf+RT	0.0	4.928e-35	210.0	0.0
Majority	nf+RT+conf	0.0	4.928e-35	210.0	0.0
Dictator	RT	3640.0	1.246e-14	201.0	251.184
Dictator	RT+conf	1492.0	4.194e-25	202.0	102.958
Dictator	nf+RT	1303.5	2.577e-25	198.0	89.950
Dictator	nf+RT+conf	1033.0	5.771e-28	204.0	71.284
RT	RT+conf	1850.5	8.700e-21	190.0	127.697
RT	nf+RT	998.5	1.080e-24	185.0	68.903
RT	nf+RT+conf	858.0	1.622e-27	196.0	59.208
RT+conf	nf+RT	6312.0	0.2608	167.0	435.569
RT+conf	nf+RT+conf	2069.0	5.181e-12	154.0	142.774
nf+RT	nf+RT+conf	2242.0	1.045e-11	156.0	154.713

Table S2d. Statistical results of pairwise comparisons between different decision support system using two-tailed Wilcoxon signed rank test with Holm-Bonferonni adjustments for experiment 2 and group size 5

**Method1**	**Method2**	**Test statistics**	**Corrected P-value**	**DoF**	**Effect size**
Majority	Dictator	6287.0	5.566e-13	237.0	396.044
Majority	RT	0.0	1.172e-41	250.0	0.0
Majority	RT+conf	0.0	1.172e-41	250.0	0.0
Majority	nf+RT	0.0	6.326e-42	252.0	0.0
Majority	nf+RT+conf	0.0	6.326e-42	252.0	0.0
Dictator	RT	4047.5	7.159e-21	238.0	254.968
Dictator	RT+conf	2425.5	3.152e-27	236.0	152.792
Dictator	nf+RT	2574.0	3.152e-27	239.0	162.147
Dictator	nf+RT+conf	1931.5	1.605e-30	241.0	121.673
RT	RT+conf	3722.0	2.046e-15	206.0	234.464
RT	nf+RT	2983.5	3.563e-16	195.0	187.943
RT	nf+RT+conf	1236.0	3.679e-27	205.0	77.861
RT+conf	nf+RT	7909.0	0.2830	186.0	498.220
RT+conf	nf+RT+conf	2811.0	1.153e-12	174.0	177.076
nf+RT	nf+RT+conf	3114.0	1.334e-09	166.0	196.164

Table S2e. Statistical results of pairwise comparisons between different decision support system using two-tailed Wilcoxon signed rank test with Holm-Bonferonni adjustments for experiment 2 and group size 6

**Method1**	**Method2**	**T-statistics**	**Corrected P-value**	**DoF**	**Effect size**
Majority	Dictator	2125.5	4.045e-22	204.0	146.673
Majority	RT	0.0	4.907e-35	210.0	0.0
Majority	RT+conf	0.0	4.907e-35	210.0	0.0
Majority	nf+RT	0.0	4.907e-35	210.0	0.0
Majority	nf+RT+conf	0.0	4.907e-35	210.0	0.0
Dictator	RT	1804.5	7.301e-21	189.0	124.522
Dictator	RT+conf	392.0	3.692e-31	200.0	27.051
Dictator	nf+RT	1134.5	8.427e-27	200.0	78.288
Dictator	nf+RT+conf	607.0	7.754e-31	205.0	41.887
RT	RT+conf	934.5	6.161e-21	163.0	64.487
RT	nf+RT	1428.0	2.783e-15	151.0	98.541
RT	nf+RT+conf	194.5	1.312e-27	171.0	13.422
RT+conf	nf+RT	3345.0	0.00031	144.0	230.827
RT+conf	nf+RT+conf	3164.0	0.1293	122.0	218.337
nf+RT	nf+RT+conf	490.0	3.386e-16	118.0	33.813

Table S2f. Statistical results of pairwise comparisons between different decision support system using two-tailed Wilcoxon signed rank test with Holm-Bonferonni adjustments for experiment 2 and group size 7

**Method1**	**Method2**	**Test statistics**	**Corrected P-value**	**DoF**	**Effect size**
Majority	Dictator	1307.5	5.784e-06	106.0	119.358
Majority	RT	0.0	3.887e-20	119.0	0.0
Majority	RT+conf	0.0	2.860e-20	120.0	0.0
Majority	nf+RT	0.0	3.887e-20	119.0	0.0
Majority	nf+RT+conf	0.0	3.887e-20	119.0	0.0
Dictator	RT	301.5	1.348e-15	110.0	27.523
Dictator	RT+conf	55.0	3.615e-19	115.0	5.021
Dictator	nf+RT	271.0	1.163e-16	114.0	24.739
Dictator	nf+RT+conf	78.0	1.161e-19	119.0	7.120
RT	RT+conf	355.5	1.244e-07	73.0	32.452
RT	nf+RT	899.5	0.0035	77.0	82.113
RT	nf+RT+conf	174.0	2.608e-11	79.0	15.884
RT+conf	nf+RT	847.0	0.1313	65.0	77.320
RT+conf	nf+RT+conf	437.5	0.00019	63.0	39.938
nf+RT	nf+RT+conf	77.5	6.397e-09	57.0	7.075

Table S2g. Statistical results of pairwise comparisons between different decision support system using two-tailed Wilcoxon signed rank test with Holm-Bonferonni adjustments for experiment 2 and group size 8

**Method1**	**Method2**	**Test statistics**	**Corrected P-value**	**DoF**	**Effect size**
Majority	Dictator	78.5	2.529e-05	41.0	11.702
Majority	RT	0.0	6.160e-08	45.0	0.0
Majority	RT+conf	0.0	6.160e-08	45.0	0.0
Majority	nf+RT	0.0	6.160e-08	45.0	0.0
Majority	nf+RT+conf	0.0	6.160e-08	45.0	0.0
Dictator	RT	7.0	1.603e-07	42.0	1.043
Dictator	RT+conf	0.0	3.546e-08	45.0	0.0
Dictator	nf+RT	0.0	4.517e-08	45.0	0.0
Dictator	nf+RT+conf	0.0	3.546e-08	45.0	0.0
RT	RT+conf	0.0	2.658e-06	32.0	0.0
RT	nf+RT	0.0	0.00019	21.0	0.0
RT	nf+RT+conf	0.0	1.942e-06	33.0	0.0
RT+conf	nf+RT	91.0	0.0059	29.0	13.565
RT+conf	nf+RT+conf	52.5	0.3839	16.0	7.826
nf+RT	nf+RT+conf	20.0	0.0017	19.0	2.981
